# Supplementary material for: Tooth fracture frequency in gray wolves reflects prey availability
Source: eLife. 2019 Sep 24;8:e48628. doi: 10.7554/eLife.48628 (PMC6759352; doi:10.7554/eLife.48628)
Supplement: Supplementary file 1. [file elife-48628-supp1.docx]

**Tooth Fracture Frequency in Gray Wolves Reflects Prey Availability**

Blaire Van Valkenburgh^1^**,** Rolf O. Peterson^2^, Douglas W. Smith^3^, Daniel R. Stahler^3^, and John Vucetich^2^

Supplementary Information

Table S1. List of the gray wolf skull samples included as “Other NA Wolves” indicating the number of individual skulls examined for each (N skulls), the total number of teeth (N teeth), the percent of individuals with at least one tooth broken, the percent of total teeth broken both with canine teeth included (% broken teeth) and excluded. Specimens are part of the US Museum of Natural History collections and labeled as the following subspecies: Alaska, *C. lupus pambasileus*; Canada/Idaho, *C. l. irremotus*; and New Mexico/Texas, *C. l. mongollensis/mostrabilis.*

| SAMPLE | N  skulls | N  (teeth) | % individuals w/>1 brkn tooth | %  broken teeth | % broken teeth with canine teeth excluded |
| --- | --- | --- | --- | --- | --- |
|  |  |  |  |  |  |
| ALASKA | 74 | 2910 | 48.7 | 4.3 | 3.6 |
| CANADA/IDAHO | 66 | 2459 | 32 | 2 | 1.8 |
| NEW MEXICO/TEXAS | 83 | 3250 | 57.8 | 3.8 | 3.8 |

Table S2. Percentage of individuals within each sample assigned to each of the three wear stages and the percent of tooth fracture (number broken/number observed multiplied by 100) for each of the five samples. Sample sizes for each tooth position in parentheses. Wear stages: S, slight; M, moderate, H, heavy. Teeth: I, incisors; C, canines, P, premolars, CR, carnassials, M. post-carnassial molars.

| SAMPLE | % s | % m | | %  h | | I | | C | PM | CR | M |
| --- | --- | --- | --- | --- | --- | --- | --- | --- | --- | --- | --- |
|  |  | |  | |  | |  |  |  |  |  |
| Isle Royale | 2 | | 63 | | 36 | | 5.5 (433) | 20 (178) | 10 (611) | 11.1 (217) | 4.2 (377) |
| SCANDINAVIA | 64 | | 29 | | 7 | | 1.8 (1079) | 3.7 (355) | 1 (1272) | 4 (372) | 0.7 (716) |
| Other NA wolves | 61 | | 33 | | 6 | | 2.3 (2479) | 6.3 (791) | 2.3 (2802) | 3 (878) | 1.4 (1669) |
|  |  | |  | |  | |  |  |  |  |  |
| YNP, 96-06 | 56 | | 39 | | 5 | | 2.2 (851) | 7.4 (272) | 1.1 (972) | 0.7 (306) | 0 (561) |
| YNP, 07-16 | 29 | | 48 | | 23 | | 6.8 (824) | 11.2 (294) | 3.4 (990) | 5.3 (304) | 1 (582) |

Table S3. Data on skeletal elements remaining at adult elk at wolf kills in YNP between 1997 and 2016. Maximum N is the total number of each element that could have been observed over the sampling period based on 2372 documented kills. The average % remaining at kill sites each year is the mean of the annual estimates of the number of elements present divided by the expected total for each element

| SKELETAL ELEMENT | N per skeleton | MAXIMUM N | AVG. % REMAINING AT KILL EACH YEAR | RANGE | SD |
| --- | --- | --- | --- | --- | --- |
|  |  |  |  |  |  |
| dentary | 2 | 4744 | 90 | 80-99 | 4.7 |
| vertebra | 26 | 61672 | 85 | 68-100 | 8.6 |
| femur | 2 | 4744 | 82 | 66-96 | 8.3 |
| tibia | 2 | 4744 | 84 | 71-94 | 6.7 |
| metatarsus | 2 | 4744 | 84 | 70-94 | 6.6 |
| scapula | 2 | 4744 | 63 | 50-92 | 9.4 |
| humerus | 2 | 4744 | 72 | 49-92 | 11.5 |
| radius + ulna | 2 | 4744 | 73 | 51-91 | 10.5 |
| metacarpus | 2 | 4744 | 73 | 53-91 | 10.3 |

Table S4. Skeletal representation per element per year. VERT, vertebra; MT, metatarsus; SCAP, scapula; HUM, humerus RAD/ULNA, radius and ulna; MC, metacarpus. For each element, the value represents the total number of elements present divided by the total number of expected elements for a given year. The annual average of these values (and standard deviation) are shown as well.

| YEAR | DENTARY | VERT | FEMUR | TIBIA | MT | SCAP | HUM | RAD/ULNA | MC | MEAN | SD |
| --- | --- | --- | --- | --- | --- | --- | --- | --- | --- | --- | --- |
| 1997 | 0.97 | 0.94 | 0.95 | 0.93 | 0.94 | 0.90 | 0.91 | 0.90 | 0.90 | 0.93 | 0.024 |
| 1998 | 0.85 | 0.71 | 0.82 | 0.84 | 0.85 | 0.65 | 0.75 | 0.76 | 0.76 | 0.78 | 0.069 |
| 1999 | 0.87 | 0.84 | 0.84 | 0.83 | 0.84 | 0.67 | 0.72 | 0.72 | 0.72 | 0.78 | 0.074 |
| 2000 | 0.81 | 0.94 | 0.81 | 0.80 | 0.80 | 0.60 | 0.68 | 0.70 | 0.69 | 0.73 | 0.102 |
| 2001 | 0.88 | 0.84 | 0.72 | 0.76 | 0.77 | 0.57 | 0.55 | 0.61 | 0.62 | 0.70 | 0.120 |
| 2002 | 0.81 | 0.79 | 0.80 | 0.82 | 0.82 | 0.59 | 0.70 | 0.72 | 0.72 | 0.75 | 0.076 |
| 2003 | 0.91 | 0.92 | 0.89 | 0.88 | 0.88 | 0.66 | 0.82 | 0.82 | 0.82 | 0.84 | 0.079 |
| 2004 | 0.85 | 0.81 | 0.80 | 0.81 | 0.81 | 0.56 | 0.64 | 0.66 | 0.66 | 0.73 | 0.104 |
| 2005 | 0.89 | 0.86 | 0.83 | 0.82 | 0.82 | 0.61 | 0.72 | 0.72 | 0.72 | 0.78 | 0.088 |
| 2006 | 0.95 | 0.92 | 0.94 | 0.94 | 0.93 | 0.78 | 0.90 | 0.91 | 0.90 | 0.91 | 0.052 |
|  |  |  |  |  |  |  |  |  |  |  |  |
| 2007 | 0.96 | 0.86 | 0.86 | 0.85 | 0.85 | 0.61 | 0.68 | 0.68 | 0.68 | 0.78 | 0.120 |
| 2008 | 0.91 | 0.86 | 0.87 | 0.86 | 0.85 | 0.65 | 0.79 | 0.80 | 0.80 | 0.82 | 0.075 |
| 2009 | 0.86 | 0.84 | 0.77 | 0.80 | 0.80 | 0.57 | 0.69 | 0.70 | 0.70 | 0.75 | 0.091 |
| 2010 | 0.85 | 1.00 | 0.72 | 0.78 | 0.79 | 0.52 | 0.61 | 0.62 | 0.62 | 0.72 | 0.148 |
| 2011 | 0.92 | 0.92 | 0.86 | 0.85 | 0.86 | 0.65 | 0.75 | 0.74 | 0.74 | 0.81 | 0.093 |
| 2012 | 0.90 | 0.84 | 0.72 | 0.76 | 0.76 | 0.54 | 0.60 | 0.60 | 0.60 | 0.70 | 0.123 |
| 2013 | 0.87 | 0.81 | 0.77 | 0.76 | 0.75 | 0.62 | 0.63 | 0.63 | 0.64 | 0.72 | 0.090 |
| 2014 | 0.86 | 0.86 | 0.81 | 0.82 | 0.81 | 0.63 | 0.67 | 0.68 | 0.67 | 0.76 | 0.090 |
| 2015 | 0.78 | 0.71 | 0.71 | 0.68 | 0.69 | 0.59 | 0.56 | 0.60 | 0.59 | 0.66 | 0.074 |
| 2016 | 0.92 | 0.74 | 0.68 | 0.73 | 0.72 | 0.50 | 0.50 | 0.51 | 0.52 | 0.65 | 0.146 |
